# DYNAMICS OF PSYCHOLOGICAL WELL-BEING ACROSS THE TRANSITION TO WIDOWHOOD

**DOI:** 10.1093/geroni/igad104.1671

**Published:** 2023-12-21

**Authors:** Lydia Li, Rita Hu, Jay Kayser, Joonyoung Cho

**Affiliations:** University of Michigan, Ann Arbor, Michigan, United States; University of Michigan, Ann Arbor, Michigan, United States; University of Michigan, Ann Arbor, Michigan, United States; University of Michigan, Ann Arbor, Michigan, United States

## Abstract

The present study examines within-person changes in psychological well-being (PWB) associated with the transition to widowhood. We analyzed 9-wave data collected annually from a probability sample of Medicare beneficiaries. The analyzed sample included married respondents at baseline (N=3,711). Five hundred ten respondents, 208 men and 202 women, became widowed in the survey period. We created variables to indicate widowhood status, the time before and the time since widowhood. Depressive symptoms measured by the PHQ9 and positive well-being assessed by a 6-items scale (e.g., My life has meaning and purpose) were dependent variables. Fixed-effect linear regression, which uses only within-person change over time in the panel data, was used. Results show that immediately after the spouse’s death, depressive symptoms increased and positive well-being decreased. There is a curvilinear relationship between time since widowhood and both depressive symptoms and positive well-being. Specifically, after the widowhood transition year, depressive symptoms began to decline, positive well-being began to increase, up to about four years after widowhood when depressive symptoms slightly rose and positive well-being slightly declined. Men and women have similar patterns. The findings suggest that right after spousal loss is challenging for older adults; some may need counseling and extra support. About two years after the spouse’s death, they return to the pre-loss level of PWB and continue improving until about four years after widowhood. Improvement in depressive symptoms and positive well-being shows older adults’ resilience and potential for personal growth after adversity.

